# β-elemene enhances the antitumor activity of erlotinib by inducing apoptosis through AMPK and MAPK pathways in TKI-resistant H1975 lung cancer cells

**DOI:** 10.7150/jca.53382

**Published:** 2021-02-22

**Authors:** Jue Wang, Cong Xu, Ying Chen, Le Shao, Ting Li, Xingxing Fan, Lili Yu, Ruonan Zhang, Bi Chen, Hongwei Chen, Xinbing Sui, Elaine Lai-Han Leung, Qibiao Wu

**Affiliations:** 1 Faculty of Chinese Medicine, Macau University of Science and Technology, Taipa, Macau, China.; 2 State Key Laboratory of Quality Research in Chinese Medicines (Macau University of Science and Technology), Taipa, Macau, China.; 3 Faculty of Medicine, Macau University of Science and Technology, Taipa, Macau, China.; 4 GCP center, the Affiliated Hospital of Nanjing University of Chinese Medicine, Jiangsu Province Hospital of Chinese Medicine, Nanjing, Jiangsu, China.; 5 The First Hospital of Hunan University of Chinese Medicine, Changsha, Hunan, China.; 6 Department of Medical Oncology, Holistic Integrative Oncology Institutes and Holistic Integrative Cancer Center of Traditional Chinese and Western Medicine, the Affiliated Hospital of Hangzhou Normal University, College of Medicine, Hangzhou Normal University, Hangzhou, Zhejiang, China.; 7 Department of Cancer Pharmacology, Holistic Integrative Pharmacy Institutes, College of Medicine, Hangzhou Normal University, Hangzhou, Zhejiang, China.; 8 School of Public Health (Shenzhen), Sun Yat-Sen University, Shenzhen, Guangdong, China.; 9 Guangdong-Hong Kong-Macao Joint Laboratory for Contaminants Exposure and Health, Guangzhou, China.; 10 University Hospital, Macau University of Science and Technology Foundation, Taipa, Macau, China.

**Keywords:** β-elemene, NSCLC, TKI-resistant, mechanisms, EGFR-mutated, AMPK, apoptosis

## Abstract

Epidermal growth factor receptor (EGFR) tyrosine kinase inhibitors (TKIs) significantly improve the outcome of non-small-cell lung cancer (NSCLC) patients with EGFR mutations, however, most TKI-treated patients will develop resistance to TKIs. β-elemene, extracted from *Curcuma aromatica* Salisb., has been widely used to treat various malignant tumors, including TKI-resistant NSCLC, but, the effects and the molecular mechanisms remain unclear. In this study, the NCI-H1975 cell line harboring double mutations L858R/T790M was treated with varying concentrations of β-elemene and/or erlotinib. The effects of β-elemene on cell proliferation, migration, apoptosis, and the expression of relevant proteins of NCI-H1975 cells were evaluated. The results revealed that β‑elemene significantly inhibited the growth, colony formation capacity, wound healing ability of NCI-H1975 cells, and improved the sensitivity of NCI-H1975 cells to erlotinib. Compared with erlotinib alone, β-elemene plus erlotinib significantly promoted the apoptosis of NCI-H1975 cells, accompanied by the down-regulated expression of P-mTOR, P-EGFR, CHOP proteins and up-regulated expression of P-AMPKα and Bax proteins. Taken together, these findings demonstrate that β-elemene suppresses the proliferation and migration of TKI-resistant H1975 cells, and enhances the antitumor activity of erlotinib by inducing apoptosis through AMPK and MAPK pathways in TKI-resistant H1975 lung cancer cells, indicating that β-elemene is a promising anti-cancer therapeutic candidate for TKI-resistant NSCLC.

## Introduction

Globally, lung cancer remains the commonest cancer and the leading cause of cancer death. Non-small cell lung cancers (NSCLC) is the most prevalent type, accounting for approximately 85% of lung cancers, and most newly diagnosed NSCLC patients are already at advanced stages 1,2,3,4 when it is often too late for surgery. For the NSCLC patients with epidermal growth factor receptor (EGFR) mutation, tyrosine kinase inhibitors (TKIs) are the first-line treatment of choice 5 and greatly improve the outcome of patients. However, most TKI-treated patients will develop resistance to TKIs which is one of the most important causes of therapeutic failure 6,7,8. Therefore, there is a pressing need for research on optimal remedies.

Apoptosis is a common pathway for TKI targeted therapy and it is regulated by p53, bcl-2, fas, ICE, mdr, and other apoptosis-related genes 9-11. Abnormal expression of these genes can lead to TKI resistance. Regulating apoptosis-related genes and their expression products can reverse TKI resistance 12. The sensitivity of cell apoptosis is mainly regulated by bcl-2 and p53 genes. The p53 protein is a factor that induces apoptosis after DNA damage and mediates cell cycle arrest by up-regulating the p21 protein. Cell cycle arrest can save time for DNA repair. On the other hand, if the intracellular damage cannot be repaired, the p53 protein promotes cell apoptosis 13. The bcl-2 gene, a member of the bcl-2 family, can block cell apoptosis without affecting cell proliferation while some members of the bcl-2 family, such as the Bax gene, can promote apoptosis. Most cancer cells have lost or no function of the p53 gene, meanwhile some cancer cells produce excessive bcl-2 protein, thus inhibiting the apoptosis of cancer cells 14,15.

β-elemene, a bioactive compound extracted from *Curcuma aromatica* Salisb. (*Curcuma wenyujin* Y.H.Chen & C.Ling) 16,17, has been widely used as an antitumor agent to treat a variety of tumors including lung cancer, liver cancer, digestive tract cancer, and bladder cancer, etc., even the patients with TKI-resistant NSCLC can also benefit from the concurrent use of β-elemene with TKIs 18-21, however, the effects of β-elemene on TKI-resistant lung cancer and the underlying mechanisms of action are largely unknown. To address these issues, we investigated the effects of β-elemene on EGFR L858R/T790M double mutation NCI-H1975 cells and the potential molecular mechanisms.

## Materials and methods

### Reagents and instruments

β-elemene was purchased from LKT Labs Co., Ltd (St. Paul, MN, USA). Erlotinib was obtained from Sigma Co., Ltd (St. Louis, MO, USA). Primary antibodies against p-mTOR (Ser2448), p-EGFR (Tyr1068), p-AMPKα (Thr172), p-Erk1/2 (Thr202/Tyr204), p-AKT (Ser473), t-mTOR, t-EGFR, t-Erk, GAPDH, t-AKT, Bax, CHOP, and PARP were purchased from Cell Signaling Technology (Danvers, MA, USA). Annexin V/PI staining dye was purchased from BD Biosciences (San Jose, CA, USA). Equipment used in this study included flow cytometry (BD Biosciences, USA), spectraMax paradigm multimode microplate reader (Molecular Devices, CA, USA), electrophoresis and transfer film equipment (Bio-Rad, Hercules, CA, USA), and high-speed centrifuge (Eppendorf, Hamburg, Germany). β-elemene and erlotinib were dissolved in dimethyl sulfoxide (DMSO) to various working concentrations when used.

### Cell lines and cell culture

The human NSCLC NCI-H1975 cell line, purchased from the American Type Culture Collection (ATCC, Manassas, VA, USA), has EGFR L858R/T790M double mutations and is resistant to erlotinib. NCI-H1975 cells were grown in monolayer culture in RPMI-1640 medium supplemented with 10% fetal bovine serum (FBS), 100 μg/mL streptomycin, 100 U/mL penicillin and were cultured at 37 °C in a humidified atmosphere containing 5% CO_2_. Exponentially growing cells were used in all experiments.

### Colony formation assay

NCI-H1975 cells were planted into six-well plates (1,000 cells/well), respectively. After attachment overnight, the cells were treated with various concentrations of β-elemene (0, 20, 40, 60, and 80 μg/mL), and the medium was replaced every 4 days. The medium was discarded when colony formation was observed. The colonies were gently washed with ice-cold PBS, fixed in 4% paraformaldehyde (PFA) for 15 min, and stained with 0.5% crystal violet (containing 0.5% crystal violet, 20% methanol, and 1% PFA in ddH_2_O) for 30 min. After the excessive crystal violet solution was removed, the colonies formed were photographed and analyzed.

### Scratch wound healing assay

NCI-H1975 cells were planted into six-well plates (5×10^5^ cells/well) and incubated overnight. When the cells were approximately 70% confluent, the confluent monolayer was scratched with the tip of a 200 mL sterile pipette. The detached cells were gently washed twice with medium and removed. The cells were treated with various concentrations of β-elemene (0, 10, 20, 30, 40, and 50 μg/mL). After additional 24 hours of growth, the cells were washed twice with PBS, and images of cell monolayers were taken with a microscope using the same configurations.

### MTT cytotoxicity assay

**Figure 2:**
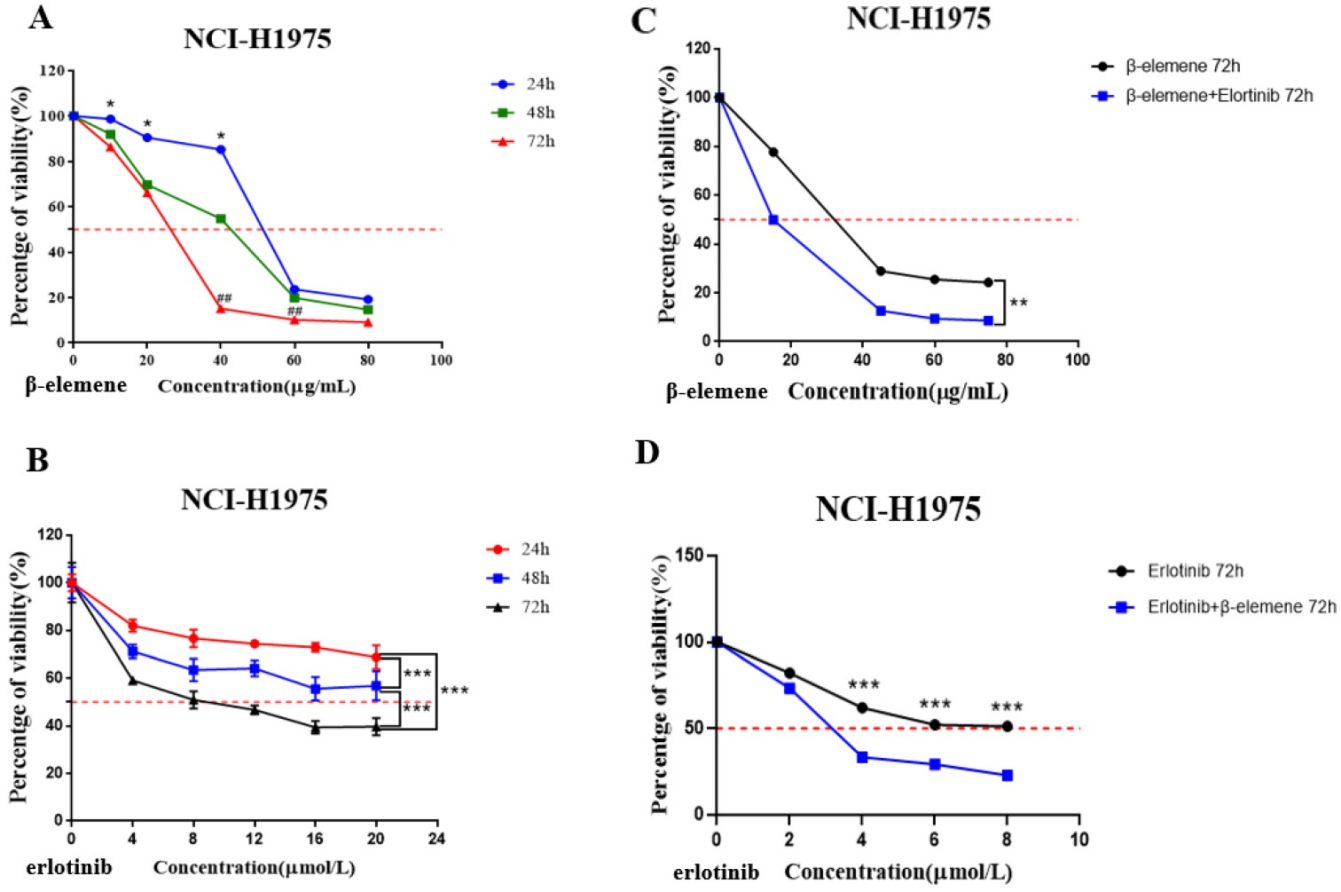
** (A)** Time- and dose-dependent growth inhibitory effects of β-elemene on NCI-H1975 cells. The cells were incubated with β-elemene for 24, 48, and 72 hrs, and the cell viability was determined by MTT assay. Data are representative of at least three independent experiments and expressed as mean ± SD; *p < 0.05, **p < 0.01, versus the cells treated for 48 hrs or 72 hrs; ^#^*p* < 0.05, ^##^*p* < 0.01, versus the cells treated for 48 hrs. **(B)** Time- and dose-dependent growth inhibitory effects of erlotinib on NCI-H1975 cells. The cells were incubated with β-elemene for 24, 48, and 72 hrs, and the cell viability was determined by MTT assay. Data are representative of at least three independent experiments and expressed as mean ± SD; *p < 0.05, **p < 0.01, ***p < 0.001. **(C)** Compared with β-elemene alone, erlotinib combined with β-elemene significantly inhibited the growth of NCI-H1975 cells. The cells were incubated with indicated concentrations of β-elemene with or without 2 μmol/L erlotinib for 72 hrs, and the cell viability was determined by MTT assay. Data are representative of at least three independent experiments and expressed as mean ± SD; *p < 0.05, **p < 0.01, erlotinib plus β-elemene versus β-elemene alone. **(D)** β-elemene significantly increased the sensitivity of NCI-H1975 cells to erlotinib. The cells were incubated with indicated concentrations of erlotinib with or without 30 µg/mL β-elemene for 72 hrs, and the cell viability was determined by MTT assay. Data are representative of at least three independent experiments and expressed as mean ± SD; *p < 0.05, **p < 0.01, ***p < 0.001, erlotinib plus β-elemene versus erlotinib alone.

NCI-H1975 cells were seeded on a 96-well microplate (5000 cells/well) and cultured overnight for cell adhesion. Two experiments were synchronously carried out to determine the sensitivity of NCI-H1975 cells to β-elemene or erlotinib, the cells were treated with a range of concentrations of β-elemene (0, 10, 20, 40, 60, and 80 μg/mL) (Fig. 2A), or erlotinib (0, 4, 8, 12, 16, and 20 μmol/L) (Fig. 2B) respectively for 24 hrs, 48 hrs, and 72 hrs, and the cell viability was assessed by 3-(4,5-dimethylthiazol-2-yl)-2,5-diphenyltetrazolium bromide (MTT) assay.

Then we carried out two experiments to investigate the synergistic effect of the combined use of β-elemene and erlotinib on NCI-H1975 cells: (a) The cells in the experiment group were treated with erlotinib (2 μmol/L) plus a range of concentrations of β-elemene (0, 15, 45, 60, and 75 μg/mL) for 72 hrs, and the control group was only treated with the same concentrations of β-elemene (Fig. 2C); (b) The cells in the experiment group were treated with a range of concentrations of erlotinib ( 0, 2, 4, 6 and 8 μmol/L) combined with β-elemene (30 μg/mL) for 72 hrs, while the control group was only treated with the same concentrations of erlotinib (Fig. 2D).

In all the above experiments, the cell viability was evaluated using a standard MTT assay. Twenty μL of MTT (5 mg/mL) solution was added to each well and incubated for 4 hrs, then culture medium was discarded and 150 μL of DMSO per well was added with oscillation for 10 min to dissolve the formazan crystals. Each dose was repeated in triplicate. Finally, we used a Tecan microplate reader (Tecan US, Inc., Morrisville, NC, USA) to measure the colorimetric intensity of the plates with a test wavelength at 570 nm and a reference wavelength at 650 nm, and calculated the cell viability as the percent change in absorbance of the treated cells divided by the absorbance of the untreated ones.

### Apoptosis assay

The NCI-H1975 cell line was seeded on a 6-well plate (2×10^5^ cells) and were serum-starved overnight. Cells were treated with erlotinib (2 μmol/L) and different concentrations of β-elemene for 24 hours. The treated cells were centrifuged after trypsin digestion. For the analysis of cell apoptosis, the cells were washed twice with cold PBS, stained with 5 μL Annexin V fluorescein and 5 μL propidium iodide (PI, 1 mg/mL) for 15 minutes at room temperature in the dark, and were then suspended in 400 μL of Annexin V binding buffer (BD Biosciences). BD FACSAria III (BD Biosciences, USA) was used to quantitatively determine the percentage of apoptotic cells.

### Western blot analysis

Protein samples were prepared from whole cell lysates and western blot was performed as follows. The cells were lysed in RIPA buffer (150 mmol/L NaCl, 50 mmol/L Tris-HCl pH 8.0, 1% Triton X-100, 1% deoxycholate, and 0.1% SDS) with protease inhibitor Roche and placed on ice for 10 minutes. The concentration of total protein extract was determined by Bio-Rad DC™ protein assay kit (Bio-Rad Laboratories, Bio-Rad Laboratories, Philadelphia, PA, USA). The same amount of total protein (40 μg) was suspended in the loading buffer, boiled at 100 °C for 5 minutes, separated by 10% SDS-PAGE, and transferred onto the nitrocellulose filter (NC) membranes (Millipore, Billerica, MA, USA). The membrane was blocked by 5% milk with Tween-20 in Tris-buffered saline (TBS) at 4 °C, and all kinds of primary antibodies were used overnight at 4 °C. The following primary antibodies were utilized: rabbit polyclonal antibodies against GAPDH (1: 1,000), phosphatase and mTOR, phosphatase and EGFR, phosphatase and Erk, phosphatase and AMPKα, Bax, poly (ADP-ribose) polymerase (PARP) (1: 1,000), and phosphatase AKT (1: 2,000), mouse monoclonal antibody against CHOP (1: 1,000). The primary antibodies were incubated overnight at 4 °C. Secondary HRP linked antibody (CST, MA, USA) was added to the membrane at 1:1000 dilution and incubated for 2 hours at room temperature after washing the membrane with TBST wash buffer three times (10 minutes/time). GAPDH was used as an internal reference protein for comparison and normalization. Immobilon western developer (Millipore, Merck KGaA, Darmstadt, Germany) and Amersham Imager 600 (General Electric, USA) were used to detect the signal intensity of the membranes.

### Statistical analysis

All data were represented as mean ± SD for three independent experiments. The inter-group differences were determined by one-way ANOVA, and then all paired chromatographic columns were compared by the Bonferroni's test. If *p* <0.05, the results were considered to be statistically significant.

## Results

### Determination of the sensitivity of NCI-H1975 cells to β-elemene or erlotinib

NCI-H1975 cells were exposed to a concentration gradient of β-elemene for 24 hrs, 48 hrs, and 72 hrs, and cell viability was assessed by MTT assay. The results clearly demonstrated that β -elemene dose- and time-dependently inhibited the cell viability. (Fig. 2A).

NCI-H1975 cells were treated with 0, 4, 8, 12,16, and 20 μmol/L of erlotinib for 24, 48, and 72 hrs, as shown in Fig. 2B, erlotinib inhibited the growth of NCI-H1975 cells in a time- and dose-dependent manner, but IC_50_ of erlotinib was extremely high: 214.5±2.32 μmol/L (48 hrs) and 9.971±0.98 μmol/L (72 hrs).

### β-elemene enhances the antitumor activity of erlotinib on TKI-resistant NCI-H1975 lung cancer cells

NCI-H1975 lung cancer cells were exposed to a range of doses of β-elemene in the absence or presence of 2 μmol/L erlotinib for 72 hrs, the results showed that compared with β-elemene alone, erlotinib combined with β-elemene significantly inhibited the growth of NCI-H1975 cells (*p* < 0.01) (Fig. 2C). After being exposed to a range of doses of erlotinib in the absence or presence of 30 μg/mL β-elemene, the β-elemene-treated cells showed significantly increased sensitivity to erlotinib (*p* <0.001) (Fig. 2D). As shown in Fig. 2C, the IC_50_ value of β-elemene in the experimental group (14.85±1.13 μg/mL) was significantly lower than that of the β-elemene group (25.85±1.33 μg/mL) (*p* < 0.01), and as shown in Fig. 2D, the IC_50_ value of erlotinib in the experimental group (3.268±1.43 μmol/L) was significantly lower than that of the control group (treated with erlotinib alone) (9.765±1.13 μmol/L) (*p* < 0.001). These results suggested that β-elemene combined with erlotinib showed a synergistic inhibitory effect on NCI-H1975 cells, β-elemene could enhance the sensitivity of NCI-H1975 cells to erlotinib.

### β-elemene inhibits cell colony formation and wound healing ability in NCI-H1975 cells

**Figure 3:**
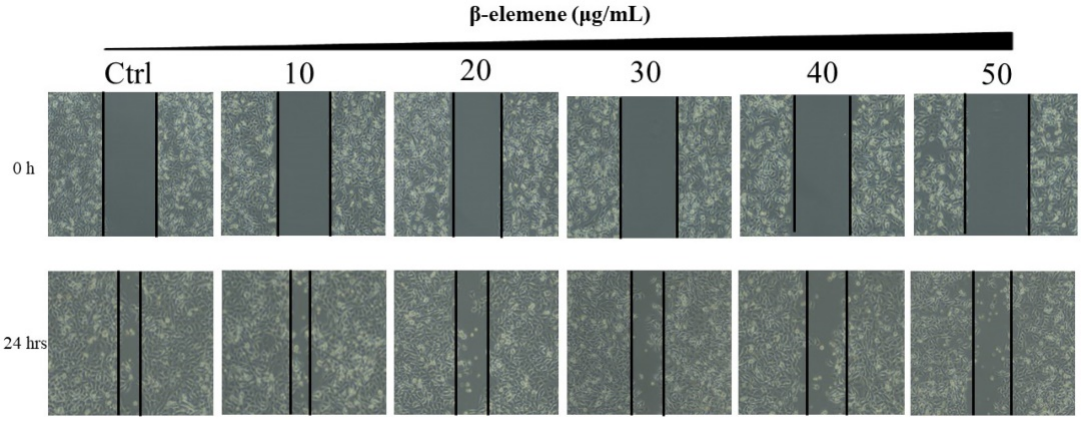
Scratch wound healing assay of NCI-H1975 cells treated with β-elemene (0, 10, 20, 30, 40 and 50 µg/mL) and monitored for 24 hrs.

**Figure 4:**
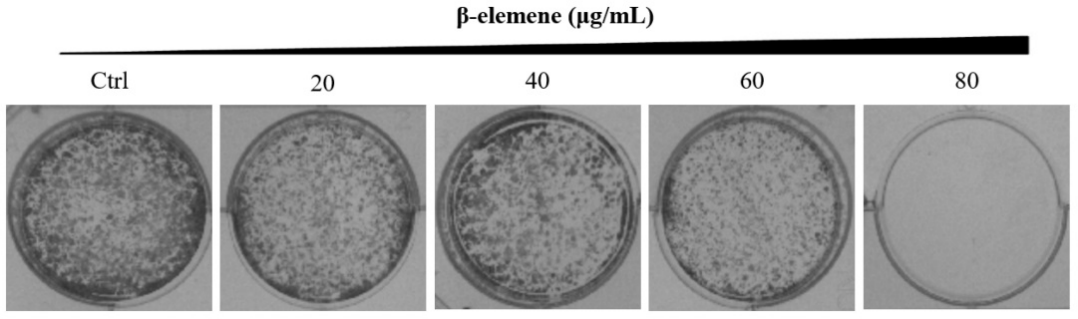
Colony formation of NCI-H1975 cells treated with β-elemene (0, 20, 40, 60, and 80 µg/mL) and monitored for 14 days. Representative photomicrographs of crystal violet-stained colonies were depicted.

Long-term colony formation assays of NCI-H1975 cells verified the growth-inhibiting effect of β-elemene. β-elemene significantly suppressed the colony formation capacities (Fig. 3) and wound healing ability (Fig. 4) of the NCI-H1975 cells in a dose-dependent manner.

### β-elemene inhibits the MAPK signaling pathway and activates the AMPK signaling pathway

**Figure 5:**
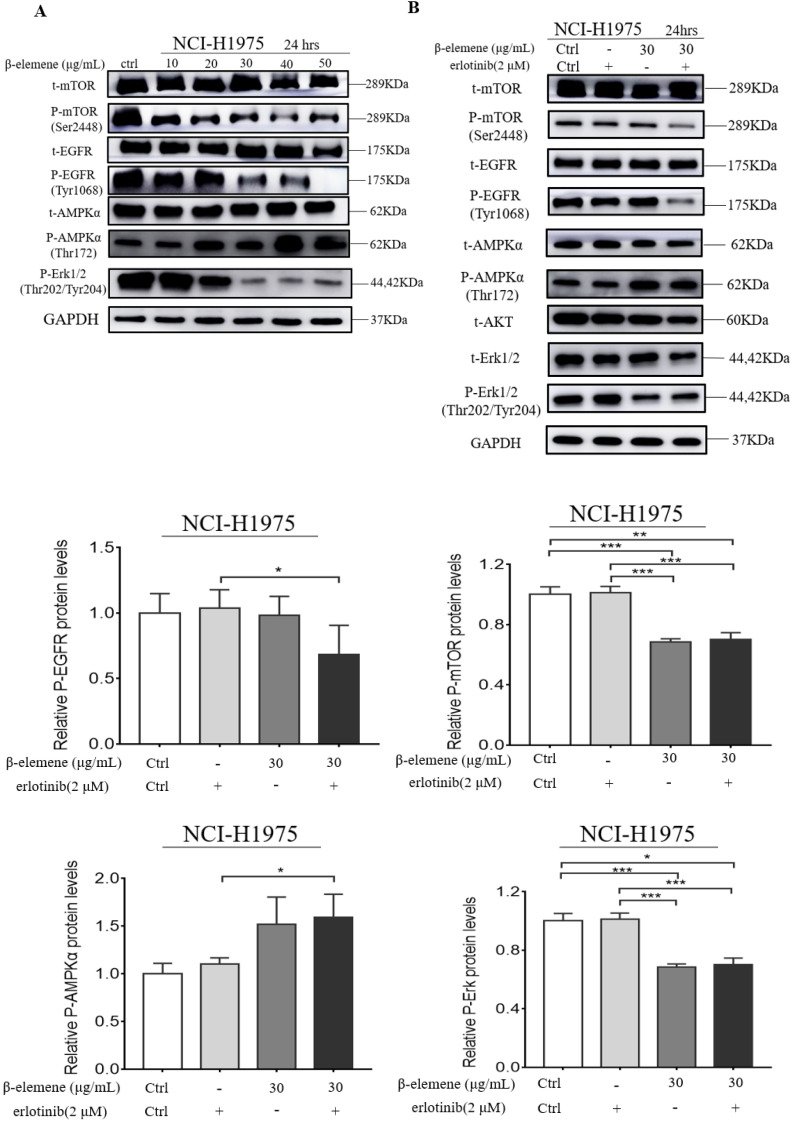
**(A)** NCI-H1975 cells were treated with β-elemene (0,10,20,30,40,50 µg/mL) for 24 hrs. **(B)** NCI-H1975 cells were treated with β-elemene (30 µg/mL) and/or Erlotinib (2 µmol/L) for 24 hrs. The expression of proteins was detected by western blot. Each experiment was repeated at least three times. Results are expressed as mean ± SD (n=3, * p <0.05, ** p <0.01, *** p <0.001).

It was reported that MAPK and AMPK were upregulated in some types of cancer, such as breast and lung cancers, and might play a key role in the modulation of cell proliferation and survival. Therefore, we investigated whether β-elemene could inhibit the MAPK signaling pathway in our cell lines by determining the phosphorylation levels of the proteins involved in this pathway, such as Erk, mTOR, and EGFR. After treatment of the cells with β-elemene for 24 hrs, we determined the phosphorylation levels of these key proteins involved in this pathway. As shown in Fig. 5, western blot results showed that β-elemene reduced the levels of phosphorylation of mTOR, EGFR, and Erk in a dose-dependent manner and activating the AMPK in NCI-H1975 cell lines, suggesting an involvement of the MAPK pathway.

### Combination use of β-elemene and erlotinib synergistically enhances the apoptosis in erlotinib-resistant NCI-H1975 cells

**Figure 6:**
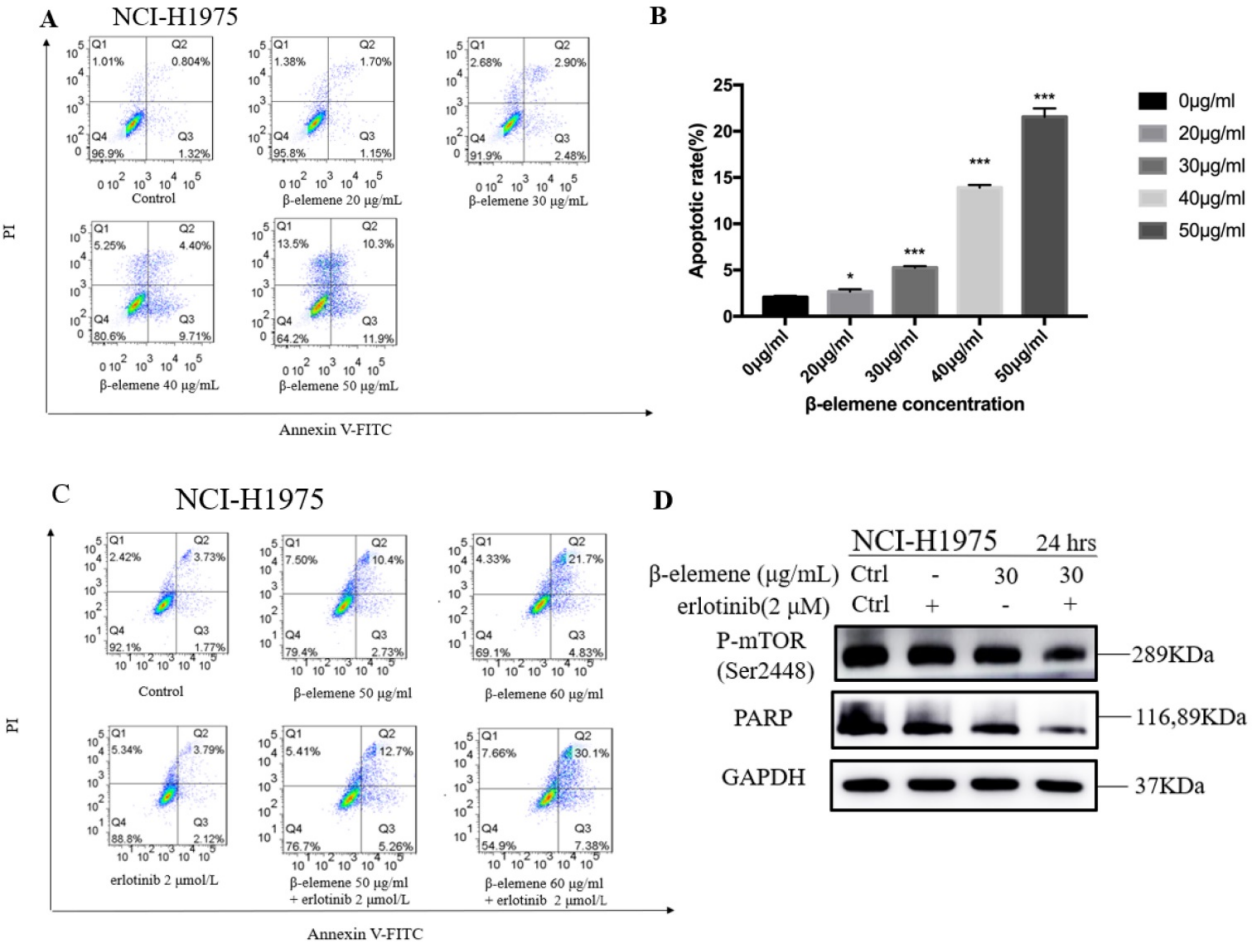
** (A)** NCI-H1975 cells were treated with β-elemene at different concentrations (0, 20, 30, 40, and 50 µg/mL) for 24 hrs. Cell apoptosis was measured by flow cytometry using Annexin V-FITC/PI staining.** (B)** Statistical analysis of the cell apoptosis rate at 24 hrs. All data are presented as mean ±SD (n=3, * p <0.05, ** p <0.01, *** p <0.001). **(C)** Annexin V-FITC/ PI staining and flow cytometry analysis of the effect of β-elemene on apoptosis of NCI-H1975 cells. **(D)**NCI-H1975 cells were treated with β-elemene (30 µg/mL) and/or erlotinib (2 µmol/L) for 24 hrs. The expression of proteins was detected by Western blot. FITC, fluorescein isothiocyanate; PI, propidium iodide.

**Figure 7:**
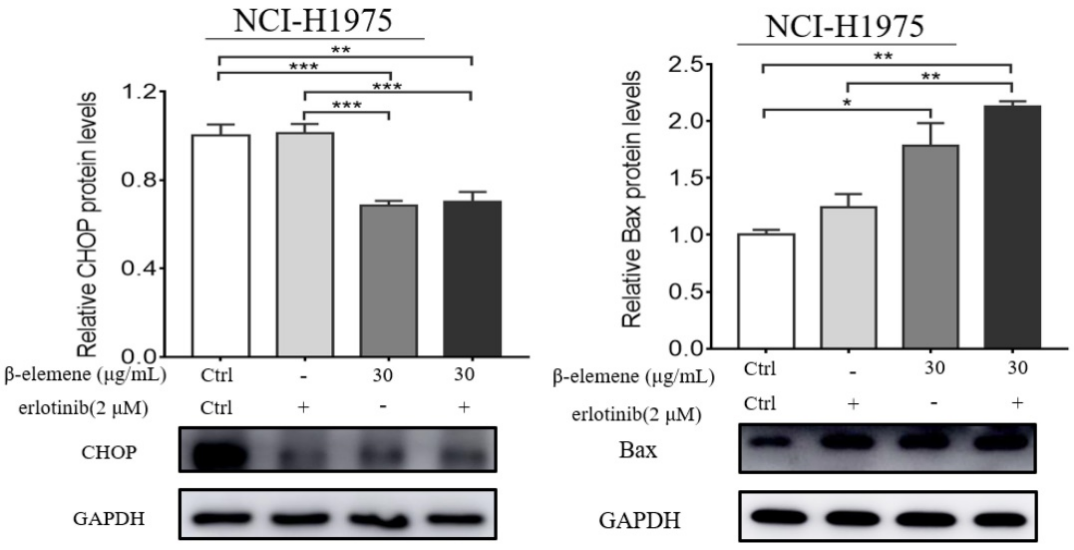
NCI-H1975 cells were treated with β-elemene (30 µg/mL) and/or Erlotinib (2 µmol/L) for 24 hrs. The expression of CHOP and Bax were detected by Western blot. Each experiment was repeated at least three times. Results are expressed as mean ± SD (n=3, * p <0.05, ** p <0.01, *** p <0.001).

To investigate the anti-cancer properties of β-elemene, we determined the level of cell apoptosis by flow cytometry using AnnexinV-FITC/propidium iodide (PI) staining. The results are shown in Fig. 6. β-elemene induced limited apoptosis in NCI-H1975 cells in low dosage, and the cells experienced extensive apoptosis with increased treatment dosage. To examine whether the increased sensitivity to erlotinib conferred by β-elemene is related to increased levels of apoptosis, we performed western blot analysis and flow cytometry following double-staining with annexin V-FITC and PI, which suggests typical changes of apoptosis. As shown in Fig. 6 and Fig. 7, western blot results showed that β-elemene inhibited the expression of CHOP, PARP while it activated Bax. The cell apoptosis rate was analyzed by flow cytometry (Fig. 6), the overall rate of apoptosis (Q2+Q3) in the erlotinib-only group was only 5.91%, indicating that treatment with 2 μmol/L erlotinib alone failed to induce NCI-H1975 cell apoptosis, but when treated with erlotinib plus β-elemene, the overall apoptosis rate increased from 5.91% to 37.48%. β-elemene plus erlotinib evidently induced NCI-H1975 apoptosis, the early apoptotic rate was 5.26%-7.38%, and the middle-late apoptotic rate was 12.7%-30.1%. These results indicated that β-elemene combined with erlotinib promoted apoptosis of NCI-H1975 cells.

## Discussion

The EGFR-mediated signaling pathways are essential for various biological processes including cancer development and progression, such as cell proliferation, adhesion, migration, differentiation, etc. 22-24, therefore, the EGFR tyrosine kinase has become an attractive target in cancer therapy, especially for NSCLC treatment. Since the first-generation EGFR-TKIs such as gefitinib and erlotinib were approved for the treatment of NSCLC in the early 2000s 25,26, TKIs have achieved some clinical benefits in the treatment of NSCLC with common EGFR activating mutations such as L858R and del E746-A750 27,28. Unfortunately, acquired resistance to TKIs is usually inevitable in almost all patients. A secondary T790M point mutation at the gatekeeper position of EGFR causes approximately 60% of NSCLC patients to develop acquired resistance after TKI treatment for a median of 10-14 months. Among the patients who have a double and coexisting mutation profile, double mutations L858R and T790M possess the highest incidence rate (9.8%) 29 and lead to the poor treatment outcome of NSCLC victims 32-35, optimal treatment strategies are desperately needed for the specific subsets of patients 36-40.

In China, Chinese medicines and their active ingredients have been widely used to treat lung cancer including TKI-resistant NSCLC^18^,^41^-^45^, and some of them have shown a synergistic inhibitory effect and lower TKIs resistance in EGFR L858R+T790M-Mutated H1975 Cells 18,32-35. β-elemene is one of these bioactive compounds and has been used to treat various cancers and has shown satisfactory efficacy with mild adverse effects 46-48. Some studies have shown that β-elemene can reverse drug resistance of cancer cells, enhance the sensitivity of cancer cells to chemotherapeutic drugs 46-48. It can induce apoptosis in lung cancer cells and other types of solid tumor cells 46-48, but the effects of β-elemene and its mechanisms of action against TKI-resistant L858R/T790M mutated NSCLC remain unknown.

The NCI-H1975 cell line harboring the mutations L858R and T790M 30,31 was selected for our study and treated with varying concentrations of β-elemene and/or erlotinib. The combined effects of β-elemene and erlotinib were evaluated. The results showed that β-elemene combined with erlotinib had a synergistic inhibitory effect on NCI-H1975 cells. β‑elemene significantly inhibited the growth, colony formation capacity, and wound healing ability of NCI-H1975 cells, and improved the sensitivity of NCI-H1975 cells to erlotinib. Our results indicated that β‑elemene might be a potential adjunctive treatment to TKI treatment for TKI-resistant NSCLC with the mutations L858R and T790M.

The MAPK pathway has been regarded as a crucial drug target for the treatment of cancer, including NSCLC 49. MAPKs family members play a key role in cell proliferation and apoptosis regulation 50. The Akt/mTOR is another important signaling pathway in regulating cell proliferation and apoptosis 51,52. mTOR has been regarded as an important target for the treatment of NSCLC. In our study, we found that β-elemene plus erlotinib had a synergistic effect on the proliferation and apoptosis of NCI-H1975 cells. β-elemene exerted potent reversal effects on TKI-resistant NCI-H1975 cell line *in vitro* through down-regulation of p-mTOR, p-EGFR, p-Erk, CHOP expression, and the activation of p-AMPKα and Bax, thus increasing the sensitivity of NCI-H1975 cells to erlotinib. These findings indicated that cell apoptosis induction contributed to the β-elemene-mediated inhibition of NCI-H1975 cell proliferation, indicating that β-elemene might help to overcome TKI resistance and improve the treatment outcome of these NSCLC patients.

However, the present study has some limitations: (a) This study is a preliminary *in vitro* study, all data are from *in vitro* experiments, which do not always extrapolate to the *in vivo* setting, more studies, especially animal experiments, are still needed to further confirm our findings. (b) The present study only studied the synergistic effect of β-elemene on the antitumor activity of erlotinib in TKI-resistant H1975 lung cancer cells and did not compare the difference in the effects of β-elemene between TKI-resistant and TKI-sensitive lung cancer cells. In the future, we will further investigate the effect of β-elemene on the TKI-sensitive lung cancer cells, such as HCC827 cells.

## Conclusion

In summary, our data provide evidence that β-elemene can inhibit the proliferation and migration of NCI-H1975 cells by inducing apoptosis. The anti-tumor effect is associated with the inhibition of MAPK activity and activation of AMPKα activity. β-elemene improves the sensitivity of NCI-H1975 cells to erlotinib, indicating that β-elemene is a promising anti-cancer therapeutic candidate for TKI-resistant NSCLC. Inhibition of apoptosis might be one of the mechanisms of the anti-tumor effects of β-elemene against NCI-H1975 cells.

## Figures and Tables

**Figure 1:**
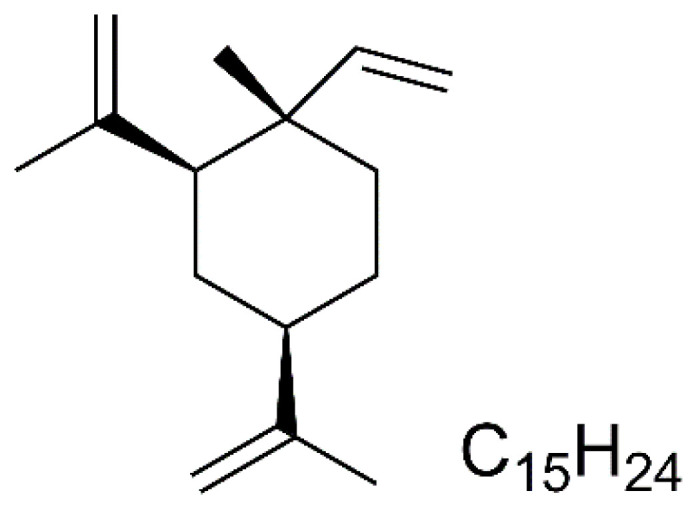
The chemical structure of β-elemene.
